# Drivers of plant diversity, community composition, functional traits, and soil processes along an alpine gradient in the central Chilean Andes

**DOI:** 10.1002/ece3.10888

**Published:** 2024-02-09

**Authors:** Lucy Schroeder, Valeria Robles, Paola Jara‐Arancio, Cathleen Lapadat, Sarah E. Hobbie, Mary T. K. Arroyo, Jeannine Cavender‐Bares

**Affiliations:** ^1^ Department of Plant and Microbial Biology University of Minnesota St. Paul Minnesota USA; ^2^ Institute of Ecology and Biodiversity (IEB) Concepción Chile; ^3^ Cape Horn International Center (CHIC) Universidad de Magallanes Punta Arenas Chile; ^4^ Departamento de Ciencias Biológicas y Departamento de Ecología y Biodiversidad, Facultad de Ciencias de la Vida Universidad Andrés Bello Santiago Chile; ^5^ Department of Ecology, Evolution and Behavior University of Minnesota St. Paul Minnesota USA; ^6^ Departamento de Ciencias Ecológicas, Facultad de Ciencias Universidad de Chile Santiago Chile

**Keywords:** alpine ecology, climatic and elevation gradients, community assembly, environmental filters, inter‐ and intraspecific trait variation, nitrogen, plant spectral traits, soil processes, taxonomic, phylogenetic, functional and spectral dimensions of biodiversity

## Abstract

High alpine regions are threatened but understudied ecosystems that harbor diverse endemic species, making them an important biome for testing the role of environmental factors in driving functional trait‐mediated community assembly processes. We tested the hypothesis that plant community assembly along a climatic and elevation gradient is influenced by shifts in habitat suitability, which drive plant functional, phylogenetic, and spectral diversity. In a high mountain system (2400–3500 m) Región Metropolitana in the central Chilean Andes (33°S, 70°W). We surveyed vegetation and spectroscopic reflectance (400–2400 nm) to quantify taxonomic, phylogenetic, functional, and spectral diversity at five sites from 2400 to 3500 m elevation. We characterized soil attributes and processes by measuring water content, carbon and nitrogen, and net nitrogen mineralization rates. At high elevation, colder temperatures reduced available soil nitrogen, while at warmer, lower elevations, soil moisture was lower. Metrics of taxonomic, functional, and spectral alpha diversity peaked at mid‐elevations, while phylogenetic species richness was highest at low elevation. Leaf nitrogen increased with elevation at the community level and within individual species, consistent with global patterns of increasing leaf nitrogen with colder temperatures. The increase in leaf nitrogen, coupled with shifts in taxonomic and functional diversity associated with turnover in lineages, indicate that the ability to acquire and retain nitrogen in colder temperatures may be important in plant community assembly in this range. Such environmental filters have important implications for forecasting shifts in alpine plant communities under a warming climate.

## Graphical abstract

We tested the hypothesis that plant–soil feedbacks along a climatic and elevation gradient influence plant community assembly through shifts in habitat suitability, which drive plant functional, phylogenetic, and spectral diversity in a high mountain system (2400–3500 m) of Región Metropolitana in the Chilean Andes (33°S, 70°W). We found that leaf nitrogen increased with increasing elevation, and taxonomic and functional diversity associated with turnover in lineages peaked at mid‐elevations, indicating that the ability to acquire and retain nitrogen in colder temperatures may be important in plant community assembly in this range. Such environmental filters have important implications for forecasting shifts in alpine plant communities under a warming climate.

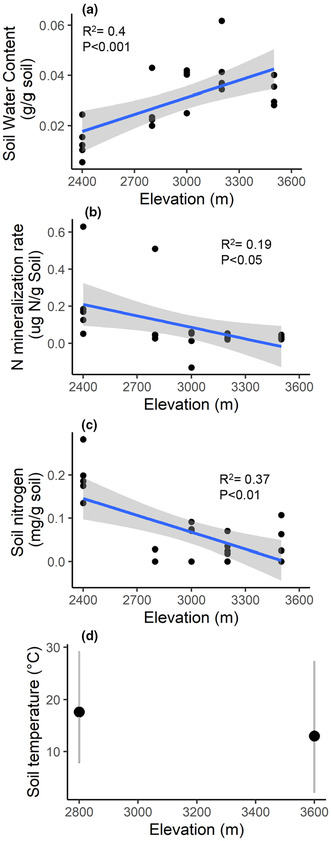

## 1 INTRODUCTION

Since von Humboldt's treatment of shifting plant communities with elevation on Mount Chimborazo (von Humboldt et al., 1824) in the Ecuadorian Andes and his phytogeographic maps of the terrestrial land surface on Earth (Johnston et al., 1848), scientists have endeavored to characterize changes in diversity across elevational, latitudinal, and other environmental gradients. Prevailing theories about community assembly posit that communities are formed through ecological and evolutionary interactions between the phenotypes and functional attributes of organisms and multiple dimensions of their environment (Bazzaz, 1991; Reich et al., 2003; Tilman, 2004; Weiher & Keddy, 1995; Woodward & Diament, 1991). Trait‐mediated assembly processes thus play a critical role in the composition and diversity of ecosystems across environmental gradients (Cavender‐Bares et al., 2004; Shipley et al., 2006). Deciphering the factors that influence species distributions remains a central goal in ecology and evolution and is paramount to understanding and modeling how ecological communities are shifting in relation to ongoing climate change.

Montane regions of the world harbor unique floras that are highly threatened by climate change (Ackerly et al., 2020; Meng et al., 2019; Wright et al., 2018). The Mediterranean climate area of central Chile in particular hosts the highest phylogenetic diversity in the country, and is a critical biodiversity hot spot to understand and protect from the effects of climate change (Cowling et al., 1996; *Biodiversidad de Chile*, 2006; Scherson et al., 2014). The purpose of the current study is to advance understanding of the abiotic drivers of plant community assembly in high‐alpine ecosystems in central Chile threatened by the effects of climate change. Our study investigates multiple dimensions of plant biodiversity and composition—including taxonomic, functional, phylogenetic, and spectral—in the high mountain environment from 2400 to 3500 m in the central Chilean Andes. We used novel and classic methods of measuring plant function and soil chemistry to discern how abiotic conditions influence plant communities to unveil potential abiotic drivers of community assembly. Taxonomic, functional, and phylogenetic dimensions of diversity may have distinct responses to varying environmental conditions along elevation gradients, and elucidating these responses can help us understand alpine community assembly (Grime, 2006; Keddy, 1992; Weiher et al., 1998). Plant species richness generally peaks at intermediate elevations along the full length of montane gradients, frequently because physiological stress at either end of the gradient is alleviated, allowing more phenotypes and species to persist (Bryant et al., 2009; Jiang et al., 2016). Species functional traits influence their tolerance to environmental factors and can reflect the ecological pressures that determine realized species assemblages (McGill et al., 2006), including along elevation gradients (Fallon & Cavender‐Bares, 2018; Read et al., 2014; Tang et al., 2022). We hypothesized that changes in aridity and temperature along the elevation gradient in the central Chilean Andes would drive shifts in plant composition that influence diversity patterns, with cold temperatures and low water availability imposing constraints at high and low elevations, respectively, such that mid‐elevations would have the highest taxonomic, functional, and spectral diversity. While it is possible that competition and facilitation likely also has a role in the expected diversity patterns, the intensity of such interactions are likely driven by the extreme environmental conditions created by high elevations, and thus we focus on environment as a primary driver of diversity (Cadotte & Tucker, 2017; Maestre et al., 2009).

We expected phylogenetic diversity to decrease with elevation, consistent with expectations under environmental filtering (Li et al., 2014; Manish, 2021; Zhang et al., 2016). Phylogenetic metrics of diversity provide insight into which lineages persist and which species co‐occur locally across the elevation gradient relative to the available species pool (Cadotte et al., 2013), which can be useful in inferring community assembly processes (Kraft & Ackerly, 2014). Phylogenetic data along with functional traits can contribute to inferences on whether adaptations to environmental niches are clustered within particular lineages and the extent to which lineages have diversified along environmental gradients (Cavender‐Bares et al., 2009; Tofts & Silvertown, 2000; Webb, 2000). Because spectral data capture both functional and phylogenetic information, we expect that they will follow similar trends, (Cavender‐Bares et al., 2017; Meireles et al., 2020; Wang & Gamon, 2019), however this expectation has not yet been tested in this system.

In high alpine Mediterranean ecosystems, above the tree line, shifting harsh abiotic environmental conditions across the gradient‐like high aridity or low temperature may be critical in limiting species and lineage distributions. Species richness and functional diversity decrease with elevation in Mediterranean alpine ecosystems, which can indicate that changes in environmental conditions limit species presence to those with particular adaptive traits (López‐Angulo et al., 2018, 2020; Moser et al., 2005) In these alpine systems, summer drought and freezing both occur, albeit to different extents along the gradient, and create gradients of harshness in opposing directions (Cavieres et al., 2006; Giménez‐Benavides et al., 2007). Drought impacts water availability and transport in the plant and can cause leaf desiccation, while freezing can damage cells due to ice formation and limit water transport by embolisms in the xylem. Both of these stress factors can limit plant species distributions and diversity (Cavender‐Bares et al., 2005). We hypothesized that shifts in plant traits both within species and communities across the gradient would correlate with increasing cold stress with increasing elevation, resulting in higher leaf mass per area (LMA) to reduce freezing damage (Ball et al., 2002; Poorter et al., 2009), and increased chlorophyll *a* concentration and leaf nitrogen concentration to maintain photosynthetic processes (Reich & Oleksyn, 2004). Increased leaf nitrogen at high elevations also helps compensate for reduced CO_2_ partial pressure at higher elevation (Körner, 2021). We also anticipated that the cooler temperatures combined with increased solar irradiance at high elevations would result in higher photoprotective stress responses through increases in xanthophyll cycle deepoxidation or increases in total xanthophyll pigment pool size, relative to the chlorophyll pool size, to avoid photodamage (Björkman, 1987; Demmig‐Adams & Adams III, 2006; Savage et al., 2009; Streb & Cornic, 2012). Cold temperatures at very high elevations may limit inorganic soil nitrogen availability due to slower microbial nitrogen mineralization rates (Fisher et al., 2013; Körner et al., 1986; Nottingham et al., 2015; Pérez‐Ramos et al., 2012). The available pool of nitrogen in the soil can affect plant functions and traits, including leaf nitrogen concentration, which is also influenced by leaf mass (Rosati et al., 2000). Varying trends in soil nitrogen across mountains globally suggest that the context of geography and local plant communities is required to understand local trends in soil nitrogen with elevation. Total soil nitrogen tends to increase with elevation and is affected by vegetation in lower altitude systems (Decker & Boerner, 2003; Garten Jr., 2004; Smith et al., 2002). In Mediterranean alpine ecosystems, soil nitrogen may influence plant diversity along the elevation gradient by selecting for species that are adapted to low nitrogen, resulting in fewer species and less functional diversity in sites with lower nitrogen availability (López‐Angulo et al., 2018, 2020). Nurse plants in these systems also play a vital role at high elevation by improving soil nutrients, increasing soil moisture, and ameliorating harsh conditions (Cavieres et al., 2006, 2007; Mihoč et al., 2016). We hypothesized that the soil resources water and nitrogen would exhibit opposing relationships with elevation: (i) soil water would increase with increasing elevation where air temperatures and evaporative demand were lower, and (ii) soil microbial activity would decline with decreasing temperature at high elevations, such that net nitrogen mineralization rates would decline with increasing elevation.

Using spectral data to estimate plant traits and diversity and linking them with soil conditions can improve understanding of potentially remote and difficult‐to‐reach alpine systems that may be threatened by climate change. Such associations in the unusual climate and abiotic conditions of high alpine Mediterranean ecosystems have yet to be tested with spectrally estimated traits and diversity.

## 2 METHODS

### 2.1 Field site

Our field sites were in the highly diverse Región Metropolitana in the Chilean Andes (33°S, 70°W). We sampled in the natural areas near Farellones, a ski resort town in colder months. In summer months, the area is used for outdoor recreation such as hiking and cycling as well as for livestock grazing. Shrubs and annual plants are mostly restricted to lower elevations, whereas cushion plants and perennial rosettes dominate in mid to high elevations above the treeline (Cavieres et al., 2000). We sampled five sites in this area located at approximately 2400, 2800, 3000, 3200, and 3500 m in elevation (see Appendix S1.1). This elevation gradient encompassed a temperature gradient with a minimum January (summer) temperature of 2.1°C at the highest elevation, and 7.8°C at the low end (Cavieres et al., 2007).

At each of the five elevations we established 10‐m transects and sampled the plant diversity using a point‐intercept method to document every individual vascular plant within a 10 cm radius at every 50 cm point along the transect (see Appendix S3.1 for a table laying out our measurements and analyses). We took care at these transects to avoid areas with high recreation traffic to capture the natural diversity of these sites. Each site had seven transects, except the highest elevation site which had eight transects to better capture the total species diversity despite the scarceness of the vegetation at this altitude. At this site, every plant within a 10 cm radius of the transect was sampled to detect rare species. In plant cover calculations, to ensure cover at this site was not overestimated using this method compared to the other sites surveyed every 50 cm, we only included plants occurring at every 50 cm along the transect (see Appendix S1.2 for visual).

### 2.2 Spectral data collection

We collected hyperspectral data for every plant sampled along the transect (every 0.5 m) using a SVC HR 1024i spectroradiometer and leaf clip foreoptic to obtain a spectral signature of each plant to estimate spectral diversity and to calculate spectral indices to estimate plant function. From each vascular plant, we removed enough leaf material to cover the foreoptic from each individual within the 10 cm radius and measured spectral reflectance within 2 h of collection. Where individuals of the same species could be distinguished, leaf material and spectra were taken from each individual. For plants with small or narrow leaves, we used more than one leaf from the same individual without overlapping to fully cover the foreoptic. A white reference was taken every ten measurements to calibrate the instrument. The spectra were compiled and trimmed to only include wavelengths from 400 to 2400 nm to remove noisy sections at the edges of the sensor's detection range with RStudio version 4.1.2 (R core Team, 2021) and the R package “spectrolab” version: 0.0.16 (Meireles et al., 2021). The spectra were vector‐normalized using the function normalize() in “spectrolab” version 0.0.16 (Meireles et al., 2021). This cleaning process resulted in a spectral dataset containing 895 bands of 1.5 nm width for each individual.

### 2.3 Spectral indices

Spectral indices associated with plant physiology or spectrally based models to predict traits were used to estimate plant function. We used reflectance bands to calculate six spectral indices related to photosynthetic function and leaf structure (see Table 1 for formulas and abbreviations). The photochemical reflectance index (PRI) is related to pools of photoprotective carotenoids (Gamon et al., 1997). Photoprotective stress responses was measured with PRI, which indicates the status of the xanthophyll deepoxidation state and total xanthophyll pigment pool size (Wong & Gamon, 2015). A low PRI value can indicate greater deepoxidation and increased plant photoprotection in response to stress. The chlorophyll *a* normalized difference index (ChlNDI) measures greenness and is sensitive to changes in chlorophyll *a* concentration (Gitelson & Merzlyak, 1994). The simple ratio of leaf mass per area (LMA) incorporates spectral absorption features of water and leaf structure and is associated with the dry matter content of the leaf (Zhang et al., 2012).

**TABLE 1:** Spectral indices used to acquire phenotypic data from leaf level hyperspectral reflectance data.

Index	Abbreviation	Equation	Citation
Simple Ratio Leaf Mass per Area	LMA	(R_860_/R_1240_)	Zhang et al. (2012)
Photochemical Reflectance Index	PRI	(R_531_ − R_570_)/(R_531_ + R_570_)	Gamon et al. (1997)
Chlorophyll *a* normalized difference index	ChlNDI	(R_750_ − R_705_)/(R_750_ + R_705_)	Gitelson and Merzlyak (1994)

### 2.4 Soil measurements

At each site along the elevation gradient, we sampled soil cores to 10 cm depth to determine the soil nitrogen and water availability. At each site, we took five samples beneath bare soil near the transects, avoiding plants and animal droppings. The bare soil samples allowed us to estimate the soil conditions without effects of the plants growing above the sampled area. The soil was sieved with a 2 mm mesh sieve to remove large rocks and organic matter. We measured the wet and oven‐dry weight of the soil samples to determine gravimetric water content. To measure total soil carbon and nitrogen concentration, the dried soil was ground to a homogenous fine powder and analyzed with a Costech ECS4010 elemental analyzer (Valencia, California, USA). We measured pH in water by mixing 10 g of the oven‐dry soil with 20 mL of nanopure water and measuring the pH of the extract with an Orion benchtop model 420A pH probe (Beverly, MA, USA). Fresh soils were shipped on ice to the University of Minnesota where we performed an initial extractions of inorganic N pools on soil subsamples in 2 M KCl within 12 days of field collection. We established room‐temperature laboratory incubations with additional subsamples in the dark, and performed extractions of inorganic N pools with 2 M KCl on incubated soils after 30 days of incubation to measure net nitrogen mineralization. Net nitrogen mineralization was calculated as the difference between initial and final extractable concentrations of ammonium (NH_4_), nitrate (NO_3_), and nitrite‐nitrogen (NO_3_‐N), which were measured colorimetrically using methods adapted from Doane and Horwáth (2003) (see Appendix S2.1 for additional detail).

### 2.5 Phylogeny construction

#### 2.5.1 Taxon sampling

DNA was obtained from leaf material of individuals collected in the field (separate material from spectral signatures) and from herbarium material stored at CONC (Herbarium of the Department Botany, University of Concepcion) and SGO (Herbarium of the National Museum of Natural History). Samples were stored in silica gel. Vouchers for field‐collected material were deposited in the herbaria CONC. We downloaded sequences for some species from GenBank (NCBI; see Appendix S3.2).The total number of taxa considered was 64. *Gingko biloba* (Ginkgoaceae) was chosen as an outgroup.

#### 2.5.2 DNA extraction, amplification, and sequencing

The DNA collected from leaf material along with GenBank sequences were used to determine relatedness and construct the phylogeny. Genomic DNA was extracted with the Dneasy Plant Kit to obtain sequences (Qiagen, Valencia, CA, USA). We amplified the DNA with PCR using the primers listed in Table S3.2 (see Appendix S2.2 for additional detail). Samples were sent to Macrogen (Seoul, South Korea) for purification and sequencing. Sequences were loaded, edited and aligned using ‘ChromasPro 2.33’ (Technelysium, Brisbane, Australia) and ‘BioEdit 7.0’ (Hall, 1999) and have been deposited in GenBank (See Appendix S3.3).We performed a combined analysis for sequences of the nuclear gene ITS and two chloroplast genes, *rbc*L and *matK*. Bayesian inference analyses were performed with MrBayes using three evolutionary models (Ronquist et al., 2012). To reconstruct the phylogeny with Bayesian inference, three partitions were used corresponding to each gene, in which evolutionary models for each genetic region were GTR + I + G in ITS; GTR + I + G in *rbc*L; and GTR + G in *matK*. Runs appeared stationary prior to 20^6^ generations, and we conservatively excluded the first 2.0 × 10^6^ generations of each run as burn‐in for the Bayesian inference. Nodes with 0.95 were considered to be robust for posterior probabilities and the predicted relationships between taxa supported (Ronquist et al., 2012). The Tracer program v1.6 (Rambaut et al., 2014) was used to visualize output parameters in order to prove stationarity and whether there are duplicated runs to converge on the same mean likelihood.

### 2.6 Leaf nitrogen quantification

Leaf tissue was collected and dried following spectral measurement. We randomly selected a subset of 233 samples, making sure to include samples of each species at each elevation for carbon and nitrogen analyses. The dried leaf tissue was ground with a Wiley mill using a 40 mesh (0.42 mm) screen, then analyzed with Costech ECS4010 elemental analyzer (Valencia, California, USA) to determine carbon and nitrogen concentrations.

### 2.7 Multiple measures of alpha diversity

#### 2.7.1 Taxonomic diversity

We calculated species richness and species evenness transect‐wise to compare the number of species at each site. We calculated Shannon diversity index in the R software package “vegan” version 2.5.7 (Oksanen et al., 2019) and divided it by the natural log of the number of species in each site to measure species evenness using the Shannon equitability index (Shannon & Weaver, 1949).

#### 2.7.2 Phylogenetic diversity

We made the phylogeny ultrametric using the nnls method with the force.ultrametric() function of “phytools” version 1.0.1 so that the branch lengths represented evolutionary time between species (Revell, 2012). Using the phylogeny and species abundance dataset, we calculated several metrics of phylogenetic diversity to compare the relatedness of species at each site. Using functions in the package “picante” version 1.8.2 (Kembel, 2010), we calculated phylogenetic species richness (PSR) using the psr() function, provides a measure of species richness that accounts for how closely are distantly related species are. PSR is calculated by multiplying the number of species in the community (in our case, each transect at each elevation is considered a community) by their evolutionary relatedness (calculated as phylogenetic species variability, PSV, based on phylogenetic branch lengths between species) (Helmus et al., 2007). Using the same package we calculated phylogenetic species evenness (PSE) with the pse() function, which incorporates species abundances with phylogenetic relatedness and richness. PSE is derived by comparing the phylogeny of each community to a hypothetical community with equal species abundances. Values of PSE are always less than or equal to one, with values close to one indicating highly even communities (Helmus et al., 2007).

#### 2.7.3 Functional diversity

Spectrally predicted leaf nitrogen and the three spectral indices (Table 1) were centered and scaled using the base R version 4.1.2 function scale() for the functional diversity calculations (Becker et al., 1998). We then created a Euclidean distance matrix from these measures. Using this distance matrix and a species abundance matrix, we calculated metrics of functional diversity of each transect using the dbFD function from the “FD” package version 1.0.12 (Laliberté et al., 2015). This function uses a distance‐based framework to calculate multidimensional functional diversity index of functional dispersion, which respectively measures the distance from the centroid of functional traits. It is important to acknowledge that these multivariate dispersion metrics introduce inherent sampling variance that may influence the true strength of their correlations with elevation.

#### 2.7.4 Spectral diversity

Spectral diversity integrates chemical, structural, and morphological diversity with light reflectance (spectra) across a range of wavelengths, providing a high‐dimensional, high‐throughput quantification of plant phenotypes (Cavender‐Bares et al., 2017; Kothari & Schweiger, 2022). Functional, phylogenetic, and spectral diversity are closely correlated in both prairie and forest systems (Laliberté et al., 2019; Schweiger et al., 2018; Wang & Gamon, 2019), making spectral measurements useful for rapidly estimating plant community diversity. We calculated spectral diversity to incorporate all the information that the full spectrum encompasses. We used the same Euclidean distance‐based method as described for the functional diversity measure, except that we used spectral reflectance values for each wavelength band as the input for trait values to calculate spectral dispersion following methods used by Schweiger et al. (2018).

### 2.8 Community composition

We calculated taxonomic, phylogenetic, functional, and spectral composition of communities using NMDS ordinations of distance matrices. For taxonomic composition, we calculated the fractional cover of each species within each of the 10 m transects by counting the abundance of each species in a transect; points without a species counted as zero. The count of each species was divided by 20, for the 20 points along the 10 m transect. Then we created a cover community matrix using the sample2matrix() function in the phylocom package (Webb et al., 2008). We calculated Euclidean distances between transects based on the abundances of each species to create an abundance‐weighted community distance matrix. For phylogenetic composition, we created abundance‐weighted community matrices for phylogenetic composition using phylogenetic mean pairwise distances between taxa in each transect using the comdist() function in the R package “picante” version 1.8.2 (Kembel, 2010). It should be noted that phylogenetic distances are estimated from trees that are themselves estimates, and therefore may have some inherent uncertainty. We calculated functional composition with community‐weighted trait Euclidean distances between transects using the function dist() in the R “stats” package version 4.1.2 (Becker et al., 1998; Borg & Groenen, 1997; Mardia et al., 1979). Similarly, we calculated spectral composition by treating each band of the short‐wave infrared (SWIR) region of the spectra (R1400‐R2400) as a trait and calculated a Euclidean distance matrix with community‐weighted means of each transect. This region is associated with water, plant biochemicals, and morphological attributes of plants, and tends to be the most phylogenetically conserved region of the spectrum (McManus et al., 2016; Ustin et al., 2009). NMDS ordinations were conducted on each of these matrices using the metaMDS() function in the R package “vegan” version 2.5.7 (Oksanen et al., 2019). For all ordinations, two dimensions were selected based on the lack of reduction in stress with higher dimensions. We fit the soil variables to the ordination using the envfit() function in the R package “vegan” version 2.5.7 to determine if communities clustered along environmental gradients (Oksanen et al., 2019). To better visualize the effect of elevation on community composition, smooth surface plots were created using the ordisurf() function from the R package “vegan” version 2.5.7 (Oksanen et al., 2019).

### 2.9 Phylogenetic signal

We compared phylogenetic signal in spectral indices and elevation using Blomberg's K (Blomberg et al., 2003). The K value in this analysis indicates the degree to which closely related species' trait values resemble each other as compared to the model. We tested for phylogenetic trait conservatism using a white noise model and a Brownian motion model following Cavender‐Bares and Reich (2012), Fontes et al. (2022), and Olalla‐Tárraga et al. (2017). Traits were averaged by species, as was elevation resulting in a mean elevation that each species was observed in. The white noise model was constructed by randomizing tips across the phylogeny over 1000 simulations and testing how the true K differed from the distribution produced by the randomization procedure. Similarly, the Brownian motion model was modeled as a “random walk” of trait evolution over 1000 simulations, bounded by positive and negative infinity. Calculating the statistic under these two models allowed us to interpret the degree of trait conservativism. Significant *p*‐values (<.05) in the white noise test indicate that the traits are more conserved than they would be by random chance. If the *p*‐value is significant under Brownian motion, it indicates that the trait is not consistent with a Brownian motion model. We used the net relatedness index (NRI) to evaluate if the communities were phylogenetically clustered or overdispersed (Webb et al., 2002). Negative values of NRI indicate more overdispersed, less phylogenetically related communities, whereas positive values indicate that communities are more closely related.

### 2.10 Statistical analysis

#### 2.10.1 Patterns of diversity with elevation

We regressed metrics of taxonomic, phylogenetic, functional, and spectral diversity against elevation and soil properties to detect the patterns of alpha diversity along our elevation gradient. To decide if the best fit was linear or polynomial, Akaike information criterion (AIC) values were calculated.

#### 2.10.2 Associations of elevation and soil properties with community composition

We conducted Mantel tests using Pearson's equations to understand the associations of the taxonomic, phylogenetic, functional, and spectral community composition with elevation and soil properties. We created community Euclidean distance matrices for each type of composition and for elevation and soil properties using the vegdist() function of the vegan package for taxonomic, functional, and spectral diversity (Oksanen et al., 2019). The phylogenetic community distance matrix was calculated using comdist() in the picante package (Kembel, 2010). This allowed us to compute dissimilarities between each transect. We ran the Mantel tests using the mantel() function in the vegan package, which compares associations between distance matrices using appropriate null models (Oksanen et al., 2019). We also ran Mantel tests to compare associations of community composition with soil properties. We then conducted partial Mantel tests using an elevation distance matrix as the control matrix, and comparing the community composition with a soil properties distance matrix using the mantel. partial() function in the vegan package. This allowed us to test the extent to which associations of plant composition with soil properties were distinct from elevation (Oksanen et al., 2019).

#### 2.10.3 Plant response to elevation and soil properties

We examined patterns of leaf traits, soil nitrogen, and soil water content at the community and species level over elevation. The traits we selected included measured percent leaf nitrogen, and spectral indices of PRI, Chl *a* NDI, and LMA, which provides information on leaf nutrient levels, stress, photosynthetic capacity, and leaf economic spectrum properties. Traits were aggregated by species. We used phylogenetic generalized least squares (PGLS) regression to examine patterns of traits with elevation and soil properties while accounting for species relatedness. We used the Holm–Bonferroni step down method to correct our *p*‐values and reduce false‐positives (Holm, 1979). We chose this method as it is the second‐most conservative *p*‐value correction method, while being uniformly as powerful as the most conservative method, the Bonferroni method, and allows for interpretation of the results but avoids inaccurate conclusions based on false‐positives.

## 3 RESULTS

### 3.1 Environmental variation with elevation

**FIGURE 1:**
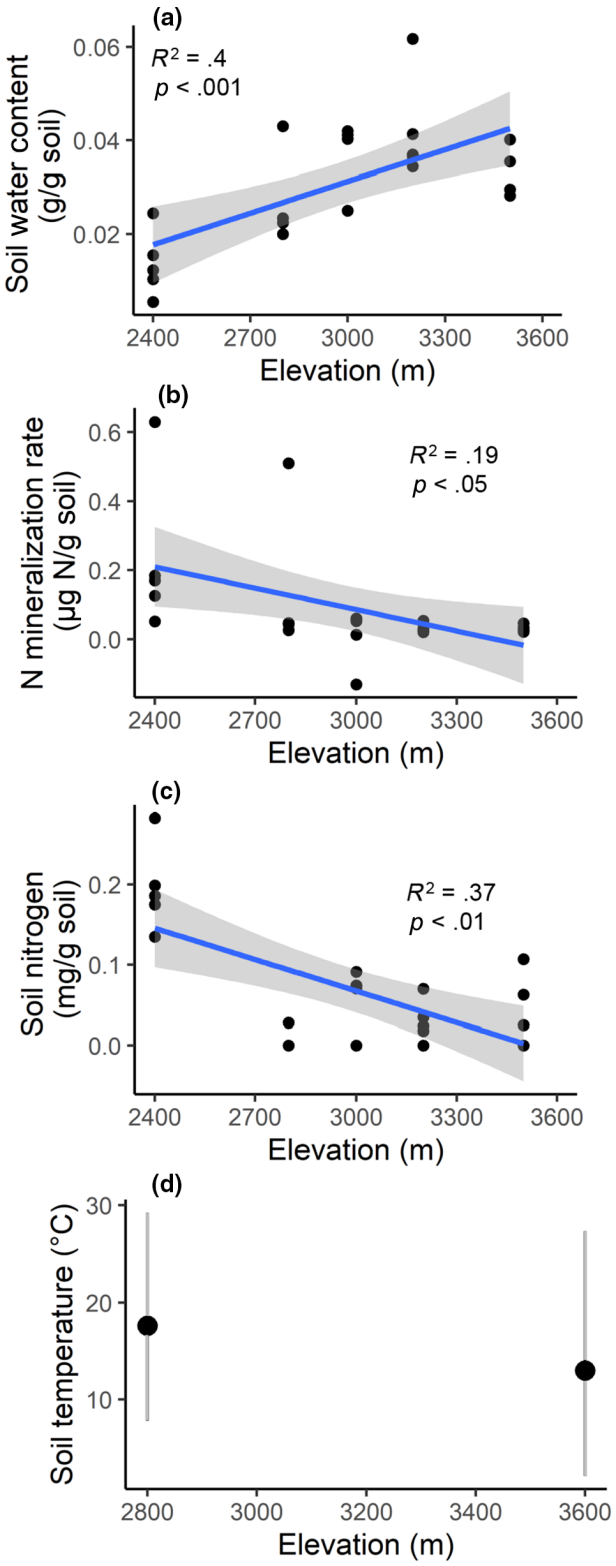
Abiotic and biotic soil variation with elevation. (a) gravimetric soil water content, (b) rate of soil net nitrogen mineralization in 30 laboratory incubations at room temperature, (c) bulk soil nitrogen concentration, and (d) January soil temperature from Cavieres et al. (2007), with vertical gray lines indicating daily range and black circles indicating daily mean temperature. Each point represents a transect sample. The best fit line is plotted in blue, with gray shading indicating its standard error.

Soil water increased linearly as elevation increased (Figure 1a). Net nitrogen mineralization rates in short‐term laboratory incubations (measured at a single, common temperature) and total soil nitrogen concentrations declined with increasing elevation (Figures 1b and 1c), as did total soil carbon concentrations (not shown). There was neither change in net nitrogen mineralization per gram of soil nitrogen with elevation nor was net nitrogen mineralization related to the soil carbon: nitrogen ratio (not shown), indicating that the observed change in net nitrogen mineralization declines in conjunction with the quantity of organic soil nitrogen available to be mineralized, rather than the availability of inorganic nitrogen.

**FIGURE 2:**
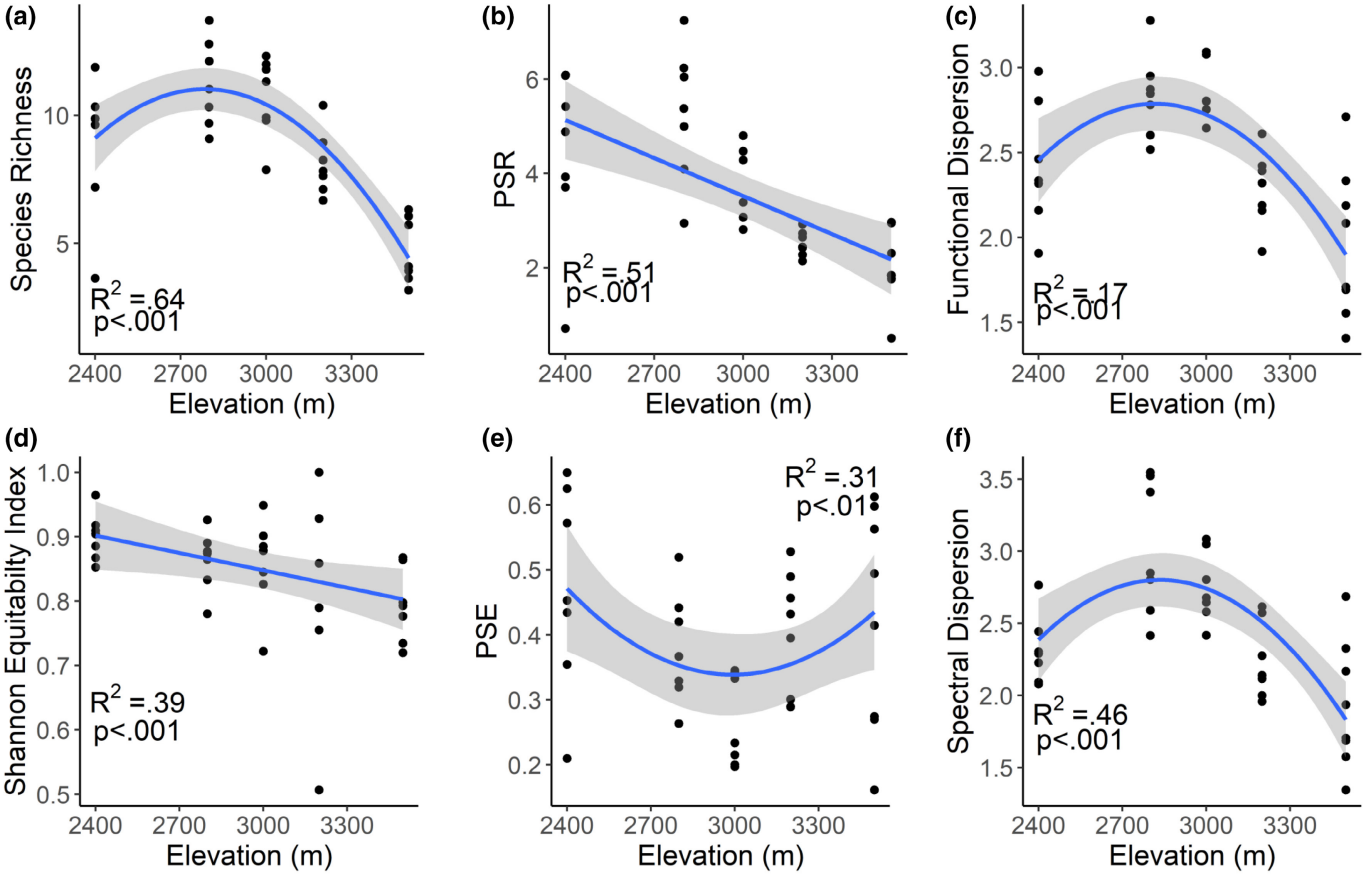
Alpha diversity across an elevation gradient, with each point indicating a transect Taxonomic diversity (a) indicated by species richness peaked at 2800 m Species evenness (Shannon equitability index) (d) decreased with elevation. Phylogenetic diversity in terms of phylogenetic species richness (PSR) (b) decreased with increasing elevation indicating that there are more phylogenetically distinct species at lower elevations, and phylogenetic species evenness (PSE) (e) indicates that the species observed were more closely related to each other at intermediate elevations. Functional dispersion (c) indicates that trait values became more similar at low and high elevations, with a peak in the middle elevation 3000 m. Similarly, spectral dispersion (f) indicates that the hyperspectral signatures were less similar at middle elevations and most similar at the highest elevation.

### 3.2 Changes in diversity indices with elevation

As hypothesized, most measures of diversity peaked mid‐way along the gradient, including species richness, functional dispersion, and spectral dispersion (Figure 2a,c,f). Species evenness (Shannon equitability index) decreased with increasing elevation (Figure 2d). Phylogenetic species richness (PSR) also decreased linearly with elevation, whereas phylogenetic species evenness (PSE) was lowest at mid‐elevations (Figure 2b,e). Linear relationships between diversity and soil properties were not significant at the *p* = .05 level.

### 3.3 Elevational shifts in community composition

**FIGURE 3:**
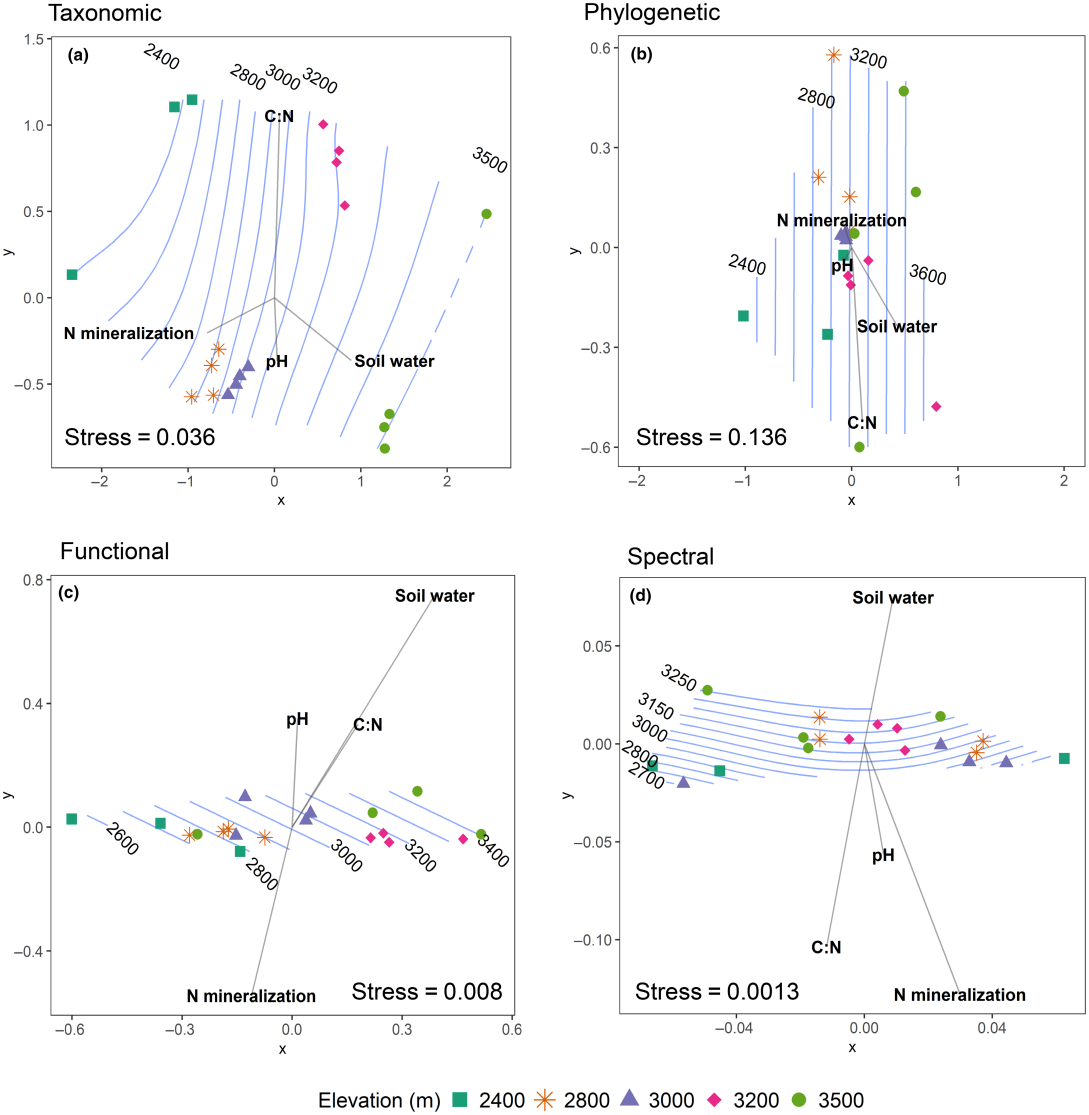
Turnover in taxonomic (a), phylogenetic (b), functional (c), and spectral (d) community composition with belowground variables across the elevation gradient using NMDS ordination. Stress values indicate the “goodness of fit” of the ordination, with values greater than 0.2 indicating poor fits. Blue contour lines are the result of the ordisurf() function fitting elevation to the ordination to create a smooth surface.

The community composition of species, phylogenetic groups, traits and spectral phenotypes shifted across the elevation gradient, revealing consistent patterns of community composition change with elevation. The highest and lowest elevations had the most distinct taxonomic communities, whereas the mid‐elevation transects (at 2800 and 3000 m) clustered near each other, indicating that the communities were similar at those elevations (Figure 3a). Communities clustered similarly dispersed across elevations when phylogenetic relatedness was considered (Figure 3b). Trait composition among sites was dispersed along the first MDS axis, though communities overlapped (Figure 3c). Spectral composition was also dispersed mostly along the first MDS axis, however the highest and lowest elevations did not distinctly separate along this axis (Figure 3d). In each of these ordinations, N mineralization and soil water changed rapidly in opposing directions. The direction of the N mineralization vector corresponded with rapid change toward low elevations, and the soil moisture vector corresponded with rapid change in the direction of higher elevations. However, the envfit analysis indicated that. The correlations of soil C:N and soil water were only significant for the phylogenetic community composition. Mantel tests showed that taxonomic, phylogenetic, functional, and spectral community composition were all significantly correlated with environmental (elevation and soil characteristics together) at the *p* = .05 level. Taxonomic (R = 0.63) and functional (R = 0.51) composition, showed the strongest relationships with environmental distances, followed by phylogenetic and spectral composition (R = 0.29 and R = 0.23, respectively). Using the Mantel test of community composition with soil properties (leaving out elevation), we found that all types of composition were lacking significant associations with soil properties at the *p* = .05 level. The results of the partial mantel tests indicate that when elevation controlled for, soil properties do not explain additional variation in plant composition, and these are not significant at the *p* = .05 level (see Table S3.4).

### 3.4 Phylogenetic analyses

The total evidence matrix for the 64 taxa included 5780 nucleotide characters (1249 ITS, 1632 *rbc*L, and 2899 *matK*), where *Gingko biloba* (Ginkgoaceae) was chosen as an outgroup. The topology of the Bayesian inference tree shows the relationships between the 64 taxa used in this study. Most taxa are well supported (>0.95; Appendix S1.3) and placed in their respective groups. The effective sample size (ESS) value was greater than 200 in a range between 406 and 17,171, indicating that the genetic regions could be combined.

### 3.5 Testing for phylogenetic signal in species traits

**FIGURE 4:**
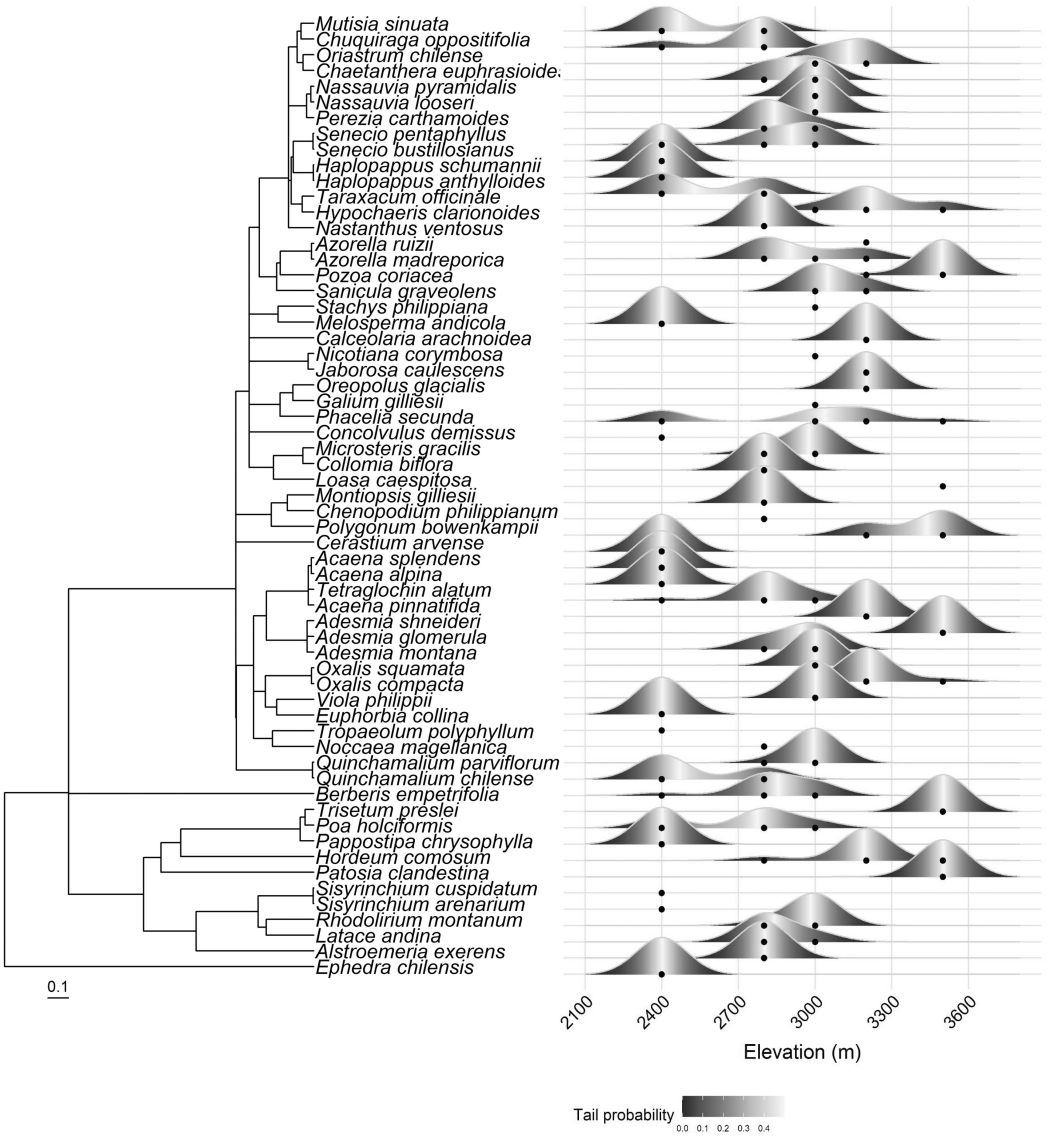
The phylogenetic tree for the species in this study alongside their elevation range distributions. The scale bar indicates the scale (0.1) of phylogenetic distance, or the relative amount of genetic change. Species occurrences at each elevation are indicated by points. The ridge plot indicates the density of each species at each elevation. Species with fewer than three occurrences are shown only with dots, no ridges. The color gradient in the ridges indicates the tail probability, with white indicating highest density and black indicating lowest density of species occurrences.

Using Blomberg's K to analyze phylogenetic signal in traits of all species in the study, we found significant (*p* < .05) nonrandom signal in spectral indices corresponding to leaf mass per area (LMA) and marginally significant (*p* < .06) nonrandom signal in leaf chlorophyll *a* using a white noise null model, suggesting some level of phylogenetic conservatism. Under a Brownian motion null model, the observed K values are statistically significant (*p* < .05), indicating that all traits are highly labile within lineages (Table 2). The *p* value of the white noise model indicates that lineages do not cluster significantly with elevation. We visualized the distribution of species and lineages with a phylogeny alongside the species distributions (Figure 4). The NRI values for the lowest elevation (2400 m) communities were highly variable but on average negative, indicating phylogenetic overdispersion in most of the low elevation communities (Appendix S1.4). The middle elevations 2800 and 3000 m averaged positive NRI values for the communities and were close to 0, indicating some phylogenetic clustering; however, the highest two elevations averaged negative. The NRI values for two transects from the 2400 m communities were statistically significant (*p* < .05).

**TABLE 2:** Phylogenetic signal of spectral indices related to various aspects of plant physiology.

	K observed	K WN *p* value	K BM *p* value	K BM mean
**LMA**	**0.260**	**0.001**	0.032	1.044
**Chl NDI**	**0.108**	**0.055**	0.001	0.972
Leaf N	0.057	0.366	0.001	0.968
PRI	0.057	0.480	0.001	1.016
Elevation	0.065	0.259	0.001	0.999

*Note*: The elevation at which each species occurs was also tested for phylogenetic conservatism. Blomberg's K values compare observed signal in traits (or elevation) to signal predicted by a white noise (WN) null model, in which species were randomized across the tips of the phylogeny, and a Brownian motion (BM) null model, in which traits were simulated to evolve along the phylogeny through a BM process. Observed K values greater than expected based on random expectation (WN) can be considered significantly phylogenetically conserved, and are shown in bold. All traits were significantly less phylogenetically conserved than expected based on a BM model of evolution.

### 3.6 Trait variation among communities and within species across environment gradient

**FIGURE 5:**
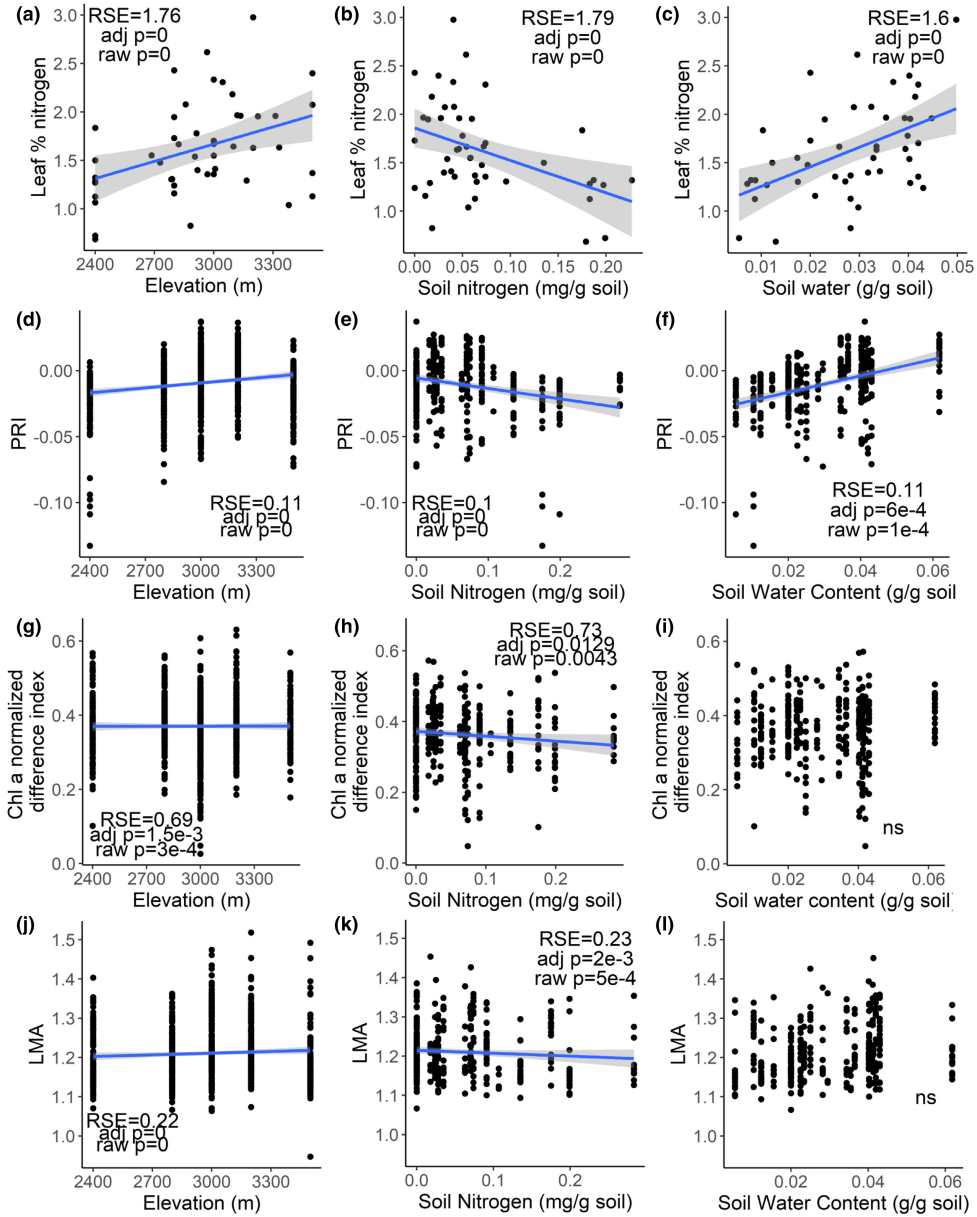
Trends in leaf nitrogen (Figure 5a–c), and spectral indices of PRI (Figure 5d–f), normalized difference chlorophyll *a* (Figure 5g–i), and LMA (Figure 5j–l) calculated from spectra with elevation and soil conditions of nitrogen and water content. Solid lines indicate significant relationships with *p* values adjusted using Holm–Bonferroni step down correction (*α* = 0.05). Nonsignificant relationships are indicated by “ns”.

Leaf nitrogen at the community level increased with increasing elevation, decreased with increasing total soil nitrogen concentration, and increased with increasing soil water (Figure 5a–c). PRI, the index of photosynthetic stress, increased with increasing elevation and soil water, and decreased with increasing total soil nitrogen (Figure 5d–f). The normalized difference index for chlorophyll *a* (Chl*a* NDI) showed chlorophyll *a* concentration significantly increasing with elevation, but decreasing with total soil nitrogen (Figure 5g–i). Leaves were more dense or thick (i.e., had higher LMA) at higher elevations but had lower LMA with higher total soil nitrogen (Figure 5j–l).

## 4 DISCUSSION

Along an elevation gradient in alpine ecosystems in the central Chilean Andes, changes in soil nitrogen availability and soil moisture appear to select for different plant functional attributes, leading to higher plant taxonomic, functional, and spectral diversity at middle elevations than at the extremes. Soil nitrogen availability declined with elevation, yet leaf nitrogen concentration increased with elevation as a consequence of both species turnover and plastic variation within species. This pattern is consistent with global patterns of increased leaf nitrogen accumulation in low temperatures, compensating for slower photosynthetic reaction rates (Körner, 2021; Oleksyn et al., 1998; Reich & Oleksyn, 2004; Woods et al., 2003).

Higher diversity at middle elevations is consistent with our hypotheses and previous findings that they represent a more accommodating environment for more species than higher or lower elevation environments (Bryant et al., 2009; Jiang et al., 2016). Differential adaptations of species to the contrasting abiotic conditions at either end of the elevation gradient explain changes in community composition. The combined patterns of plant diversity and plant function indicate that environmental filters influence plant community assembly along the elevation gradient but the nature of these filters shifts with elevation, as has been found in other montane systems (Chun & Lee, 2018; Fallon & Cavender‐Bares, 2018; Scherrer et al., 2019; Yi et al., 2020; Zhang et al., 2016). However phylogenetic diversity differed from this trend: some lineages occurred only at lower elevation sites, thus phylogenetic species richness was highest just above the tree line and decreased with increasing elevation.

### 4.1 Environmental conditions influence physiological traits along the elevation gradient

Harsh environmental conditions at both ends of the elevation gradient select for species with specific adaptations that enhance survival under those specific conditions. The low end of the elevation gradient was characterized by lower soil water availability, warmer temperatures, and more nitrogen availability, selecting for plants with thinner leaves at low elevations compared to high elevations, with high elevation leaves having more dry matter due to slower growth rates at low temperatures (Berry & Bjorkman, 1980). The reduction in net nitrogen mineralization rates with increasing elevation measured in soils incubated at a common temperature likely would be amplified in field conditions, where low temperatures at high elevations would further reduce nitrogen mineralization rates (Guntiñas et al., 2012). The reduced net nitrogen mineralization rates with increasing elevation measured here were driven by a reduction in the amount of organic soil nitrogen with increasing elevation, as evidenced by the lack of effect of elevation on nitrogen mineralization per gram of soil nitrogen. Lower plant cover at the higher elevation site likely impacts the amount of organic nitrogen inputs into the soil, leading to smaller nitrogen pools. It should be noted that at higher elevations, there were more plants of cushion form (Appendix S1.3) that facilitate the survival of other species under stressful abiotic conditions and locally increase soil nitrogen where they grow (Cavieres et al., 2007; Gavini et al., 2020; Mihoč et al., 2016). This locally increased soil nitrogen near nurse plants may allow other herb species growing at high elevations to acquire more nitrogen.

Decreased nutrient availability due to colder temperatures at the higher elevations likely drives differences in plant communities along the gradient by selecting for species with small, dense leaves (as indicated by increased LMA), and high nitrogen accumulation ability, despite low soil nitrogen. Increased leaf nitrogen and higher LMA may be linked, resulting from increased thickening of the cell walls (Onoda et al., 2017). However, the higher photosynthetic chlorophyll *a* pigment concentrations at higher elevations suggest that the leaf nitrogen accumulation at these elevations may play a role in the maintenance of photosynthetic activity. Leaf nitrogen concentration is correlated with the concentrations of chlorophyll and RuBisCO (Ellis, 1979; Evans, 1989; Luo et al., 2021). High RuBisCO concentrations help to maintain photosynthetic efficiency in cooler temperatures where biochemical reaction rates are slower (Cerqueira et al., 2019; Holaday et al., 1992).

Increased photosynthetic pigments and enzymes in plants at high elevations may also explain why photosynthetic stress responses and photoprotection, as indicated by PRI, were lower at high elevations. In addition, changes in overall plant morphology not captured by the spectra, such as dense trichomes or changes in height, may result in increased photoprotection (Molina‐Montenegro & Cavieres, 2010). At the lower elevations in our study, lower PRI indicates that photoprotective stress responses were higher, which may be caused by higher light exposure with less cloud cover and higher temperatures, increased temperature, and reduced soil water at lower elevations. Water is critical to cell expansion and growth as well as to fundamental physiological processes such as nutrient transport and photosynthesis (Lambers et al., 2009). Evidence suggests that plants from dry environments may have increased zeaxanthin levels, indicating higher xanthophyll deepoxidation rates and suggesting that plants with limited access to water increase photoprotection to help reduce photodamage (Wujeska et al., 2013).

### 4.2 Trends in diversity reflect changing environmental conditions

Alongside shifts in plant function that covaried with the changing environmental conditions along the gradient, plant community composition and diversity also changed, with highest taxonomic, functional, and spectral diversity at middle elevations. Mid‐gradient peaks in taxonomic and functional diversity indicate that the mid‐elevations supported more species and may have allowed for more niche space for species with different traits. Spectral diversity trends followed similar patterns to functional diversity. This may indicate that the leaf‐level spectral diversity we measured was more influenced by spectral detection of functional traits than by phylogenetic relatedness, likely because the spectral reflectance is detecting leaf chemistry and structure (Cavender‐Bares et al., 2017; Schweiger et al., 2018).

Phylogenetic richness and species richness patterns indicated that the pool of species at higher elevations was limited to fewer lineages. Some lineages were present only at the lowest elevation, increasing the phylogenetic species richness there. The significant Blomberg's K values for LMA and leaf chlorophyll suggest that these traits are phylogenetically conserved, although less conserved than would be expected based on Brownian Motion evolution (Table 2). Taking into account that phylogenetic richness decreased at high elevation, these traits may be especially important for survival in high alpine environments and may be shared among close relatives. However, Blomberg's K analysis indicates that lineages do not cluster significantly with elevation, in general. NRI values for the transects indicate that the plant communities at the lowest elevation are less closely related than expected (overdispersed), with few closely related species present (Appendix S1.4). At high elevations above 3200 m, we only found one graminoid species, *Hordeum comosum*, and one invasive Asteraceae species, *Taraxum officinale*. These lineages were much more diverse at lower elevations. The significant overdispersion at the lowest elevation and the lower diversity in some major lineages at higher elevations the linear trend of decreasing PSR with elevation.

Discrepancies arose in the direction of the patterns of taxonomic and phylogenetic diversity when abundance was accounted for. Species evenness decreased with increasing elevation, suggesting that at higher elevations there were a few highly abundant taxa, with other rare species in the community. The trend in phylogenetic evenness was due to uneven species abundances, driven by the presence of two highly abundant and distantly related small herb species at the 3000 m site. The mid‐gradient peaks we observed were consistent with trends in species richness in alpine environments globally (Testolin et al., 2021). Taxonomic, phylogenetic and functional trait composition clustered with elevation, indicating that communities at high and low elevations were distinct from each other and showed significant compositional change with increasing elevation. Spectral SWIR did not show clear community composition by elevation, with many communities overlapping across the gradient. This could be due to similarities in adaptations to drought and cold leading to structural similarities or similarities in water retention. Mantel tests indicate that shifts in composition are associated with elevation, but that measured soil variables do not explain additional variation, which could indicate these shifts are associated with another factor that changes across the elevation gradient, such as temperature. Considered with the environmental trends, the patterns of diversity and community composition suggest that environmental harshness may lead to turnover across the gradient by selecting for species with adaptive traits.

### 4.3 Future directions

Climate change will affect the temperature, nitrogen availability, and water availability along the elevation gradient. We will need more research to understand the community dynamics, especially dispersal, that may affect the habitat range of species restricted to higher elevations and may change community composition along the elevation gradient. Furthermore, while we focused solely on environmental filters influencing community assembly, there are a number of other filters that impact plant communities in mountains, including plant–plant interactions and dispersal. For example, nurse plant interactions can facilitate higher levels of diversity in communities at higher elevations (Cavieres et al., 2014; Gavini et al., 2020; Pashirzad et al., 2019), however competition at lower elevations can limit species from higher elevations migrating down the gradient (Choler et al., 2001). Dispersal limitation also influences plant communities, and accounting for seed size may help predict dispersal ability (Eriksson, 2000). We need a clear understanding of species dispersal abilities in addition to environmental filters, which will determine the available species pool for future community composition (Alexander et al., 2018). Differences in dispersal ability could influence plant diversity along elevation gradients by affecting which species can reach different elevations, while environmental filters impact which species survive. Furthermore, trait plasticity could allow some species to respond to changing climatic conditions. Soil and plant transplant studies should be implemented in alpine regions to determine the consequences of warming and reduced water on soil processes and the limits of plant trait plasticity.

## 5 CONCLUSION

We found evidence that environmental filters influence community assembly and diversity in the high alpine of the central Chilean Andes. While lower elevations were driest, higher elevations were the coldest and least nutrient rich. These harsh environmental conditions limit which plants can persist, based on their functional adaptations to aridity or cold. At high elevations, low nitrogen availability and temperature appear to select for species with high LMA and small leaves that can accumulate nitrogen and avert enzymatic limitation of photosynthetic rates. Intermediate elevations, where the aridity and stresses due to cold temperatures are less extreme, host the highest plant taxonomic, functional, and spectral diversity. Our results indicate that environmental stress and resource availability drive variation in plant function and influence which species and lineages can occur along the gradient, leading to changes in community composition. As the climate warms, these stresses are likely to shift upward in elevation, affecting plant communities in different ways. By using spectral data to rapidly assess plant traits, we can increase our understanding of plant community function. Deciphering how environmental conditions influence plant diversity and composition in alpine systems is an important step toward predicting how they might be affected by climate change and developing management strategies for conserving these threatened ecosystems.

## AUTHOR CONTRIBUTIONS

**Lucy Schroeder:** Conceptualization (equal); formal analysis (lead); investigation (equal); methodology (equal); writing – original draft (lead); writing – review and editing (lead). **Valeria Robles:** Data curation (supporting); investigation (supporting); writing – review and editing (supporting). **Paola Jara‐Arancio:** Funding acquisition (equal); investigation (equal); methodology (equal); writing – original draft (supporting); writing – review and editing (equal). **Cathleen Lapadat:** Conceptualization (equal); data curation (equal); methodology (equal); writing – review and editing (supporting). **Sarah E. Hobbie:** Conceptualization (equal); methodology (equal); writing – review and editing (equal). **Mary T. K. Arroyo:** Conceptualization (equal); funding acquisition (lead); supervision (equal); writing – review and editing (equal). **Jeannine Cavender‐Bares:** Conceptualization (lead); funding acquisition (lead); project administration (lead); supervision (equal); writing – review and editing (equal).

## CONFLICT OF INTEREST STATEMENT

The authors declare no conflict of interest.

### OPEN RESEARCH BADGES

This article has earned Open Data and Open Materials badges. Data and materials are available at the full data including hyperspectral data, abundances, soil data, trait data, and the phylogentic tree are available in on Dryad https://doi.org/10.5061/dryad.2ngf1vhtt. https://datadryad.org/stash/share/id6UHgzBWT6Xyb27y8btFqhIHv9DzcE6OKid3pN65gg.

## Supporting information


Appendix S1.

Appendix S2.

Appendix S3.

## Data Availability

The full data including hyperspectral data, abundances, soil data, trait data, and the phylogentic tree are available in on Dryad 10.5061/dryad.2ngf1vhtt. (Private peer review link: https://datadryad.org/stash/share/id6UHgzBWT6Xyb27y8btFqhIHv9DzcE6OKid3pN65gg).
