# Supplementary material for: Drivers of plant diversity, community composition, functional traits, and soil processes along an alpine gradient in the central Chilean Andes
Source: Ecol Evol. 2024 Feb 9;14(2):e10888. doi: 10.1002/ece3.10888 (PMC10857943; doi:10.1002/ece3.10888)
Supplement: Supplementary file 1 — Appendix S1. [file ECE3-14-e10888-s001.docx]

**Appendix S1**

**Supplemental figures**

**
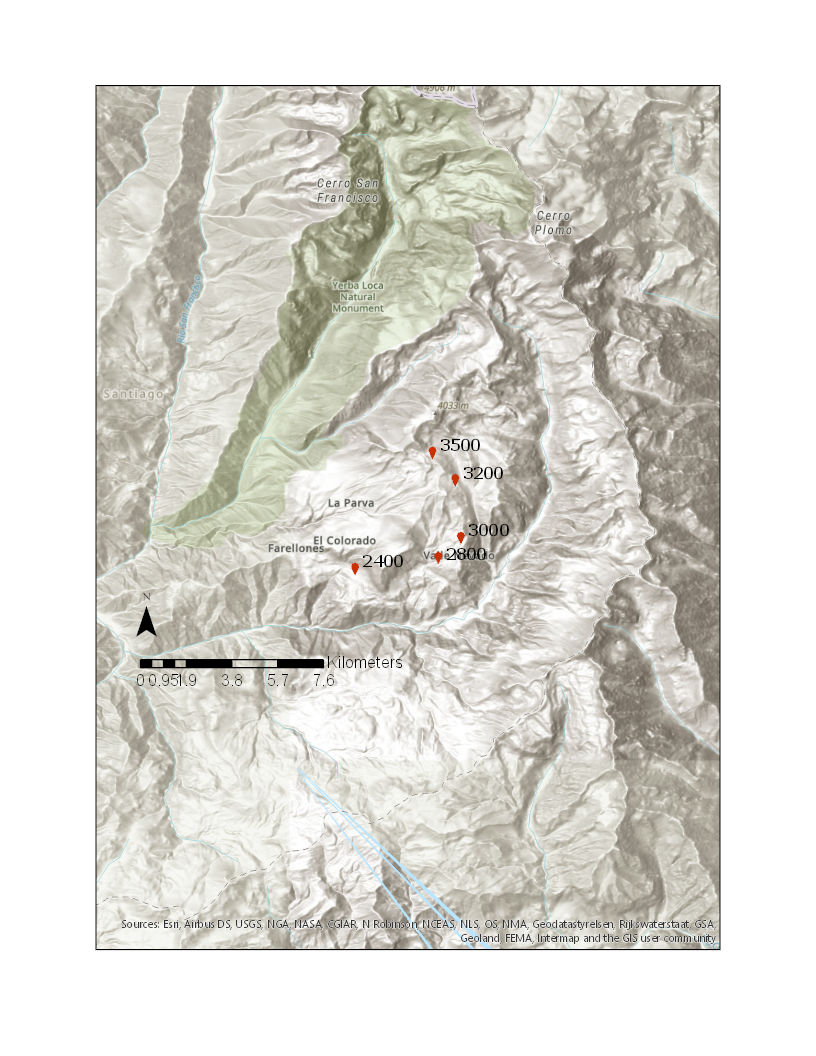
**

S1.1. Relief map of sites near Farellones used for this study.


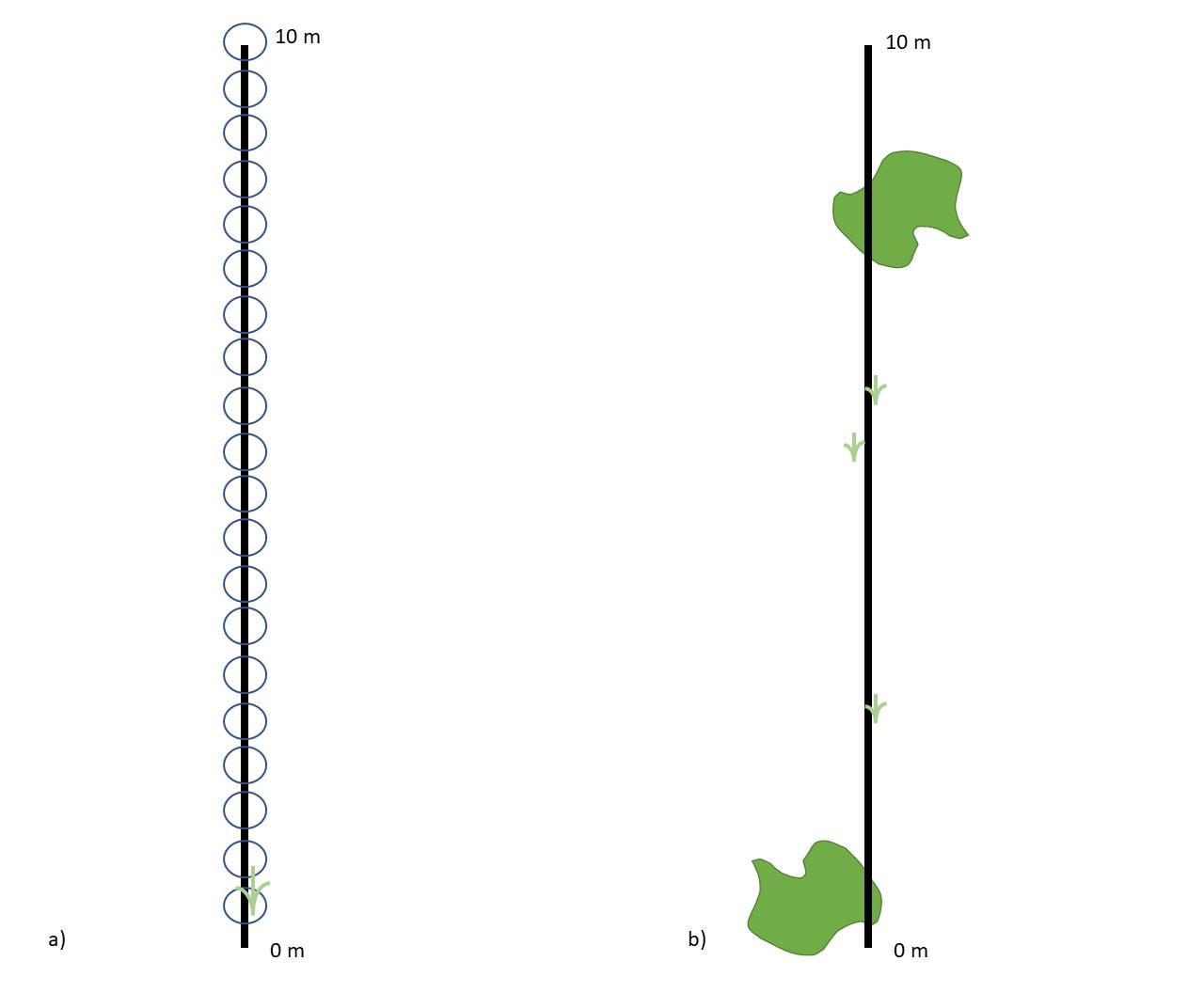


S1.2. Visual of point-intercept transect sampling. a) Typical 10 m transect used, circles represent radius of points every 0.5 m along the transect, plants within radius of point were counted in survey. b) Example of transect survey at highest elevation site. Due to the extreme sparseness of plant life, sampling every 0.5 m would not capture the full range of plant diversity so every plant along the 10 m transect was recorded.


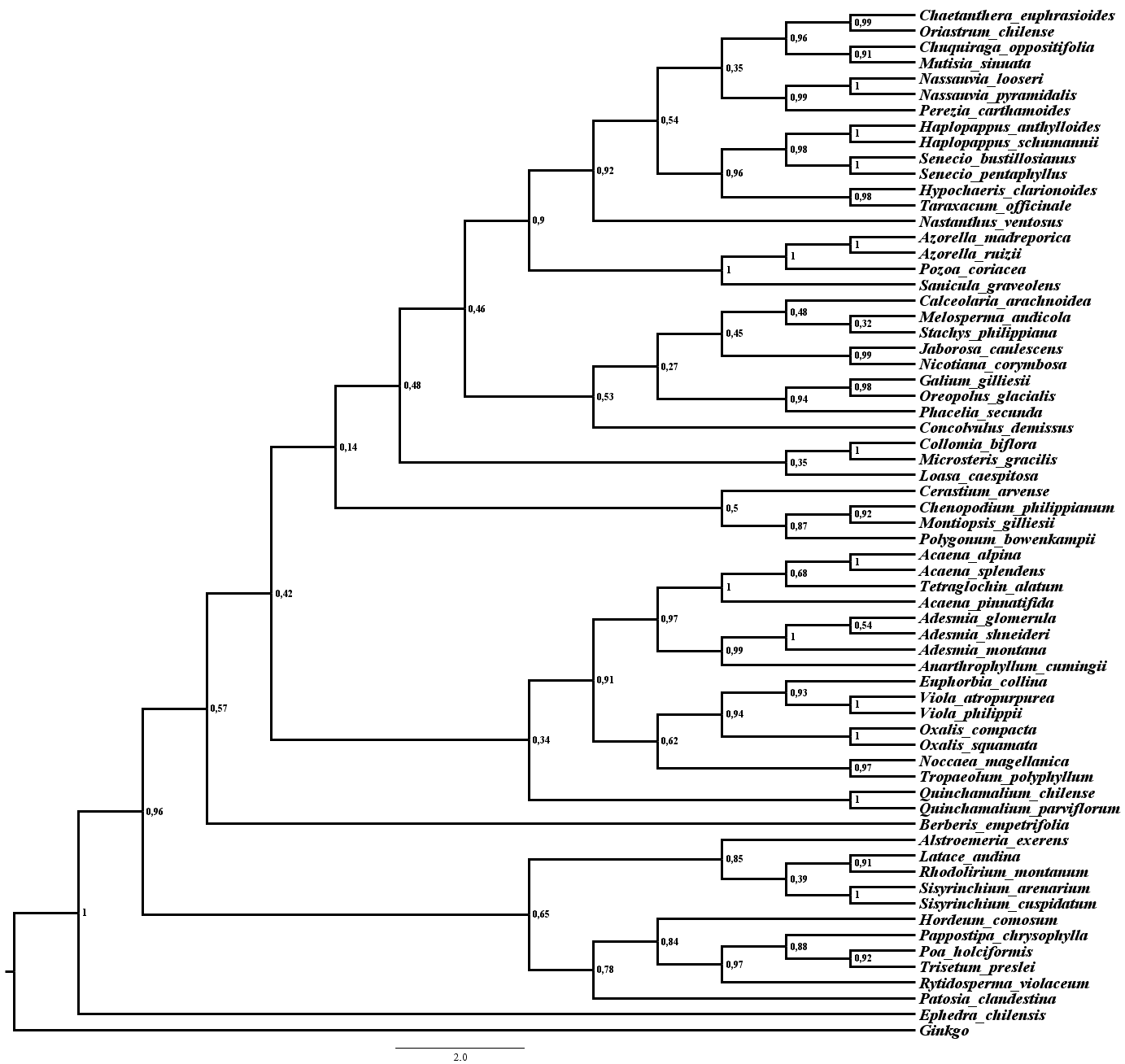


S1.3 The phylogenetic tree made with Bayesian Inference showing the relationship between the taxa used in this study. Support for the relationship is indicated at the nodes.
