# Supplementary material for: Drivers of plant diversity, community composition, functional traits, and soil processes along an alpine gradient in the central Chilean Andes
Source: Ecol Evol. 2024 Feb 9;14(2):e10888. doi: 10.1002/ece3.10888 (PMC10857943; doi:10.1002/ece3.10888)
Supplement: Supplementary file 2 — Appendix S2. [file ECE3-14-e10888-s002.docx]

**Appendix S2**

**Supplemental methods**

**S2.1 Net N mineralization detailed methods**

An initial extraction of field-moist soils using a 2M KCl solution was done within 4 days of soil collection for analysis of net nitrogen mineralization potential. The remainder was incubated for 30 days at 18 °C in the dark. After the incubation period, the soil was extracted in a 2M KCl solution, filtered with Whatman grade 42 filter paper and the extract frozen until analysis could be completed. Ammonium, nitrate, and nitrite concentrations were measured optically with microplate salicylate and sulfanilamide methods respectively, using a BioTek Synergy H1 microplate reader (Winooski, Vermont, USA).

**S2.2 PCR detailed methods**

PCR used a final volume of 30 µl, which contained 4 µl DNA (25 ng/µl), 8.35 µl distilled water, 3 µl MgCl2 (25 mM), 6 µl buffer, 2.4 µl of dNTP (1 mM), 1.8 µl of each primer (10x), 2.4 µl BSA (25 mM) and 0.25 µl GoTaq (5 U/ µl). DNA was denatured at 95°C for 5 min, followed by 35 amplification cycles of 45 s at 94°C, annealing for 1 min at 50°C, elongation for 1.5 min at 72°C and a final extension of 7 min at 72°C. For *rbc*L, DNA was denatured at 95°C for 3 min. Followed by 30 amplification cycles of 1 min at 95°C, annealing for 45 s at 50°C, elongation for 1 min at 65°C and a final extension of 5 min at 65°C, and *matK*, DNA was denatured at 95°C for 3 min. Followed by 36 amplification cycles of 30 s at 94°C, annealing for 40 s at 58°C, elongation for 1 min at 72°C and a final extension of 10 min at 72°C.
