# Supplementary material for: Drivers of plant diversity, community composition, functional traits, and soil processes along an alpine gradient in the central Chilean Andes
Source: Ecol Evol. 2024 Feb 9;14(2):e10888. doi: 10.1002/ece3.10888 (PMC10857943; doi:10.1002/ece3.10888)
Supplement: Supplementary file 3 — Appendix S3. [file ECE3-14-e10888-s003.docx]

**Appendix S3**

**Supplemental tables**

S3.1 This table lists the type of sample taken, the measurements used to create the sample, the diversity and/or spectral indices calculated from that data, and analyses performed using the calculations or measurements.

| **Sample** | **Measurements** | **Diversity indices calculated** | **Spectral indices calculated** | **Analyses performed** |
| --- | --- | --- | --- | --- |
| Hyperspectral data | spectral reflectance wavelengths from 400-2400 nm | Spectral dispersion, functional dispersion | Chlorophyll normalized difference index, Photochemical reflectance index, Simple ratio of leaf mass per area index | NMDS, phylogenetic regression of spectral indices with elevation and soil variables, Blomberg's K calculation |
| Plant survey | species count | species richness, Shannon evenness index | - | NMDS |
| Leaf material DNA | phylogenetic relatedness | phylogenetic richness, phylogenetic evenness | - | NMDS, phylogenetic regression, Blomberg's K, NRI calculation |
| Soil | Gravimetric soil water content, soil C:N ratios, soil N mineralization | - | - | NMDS envfit, phylogenetic regression with elevation and soil variables |

S3.2. Primers used to amplify and sequence rDNA and cpDNA obtained in the laboratory.

| **Primer** | **Sequence (5´-3´)** | **References** |
| --- | --- | --- |
| ITS4 | TCCTCCGCTTATTGATATGC | White et al. 1990 |
| ITS5 | GGAAGTAAAAGTCGTAACAAGG | White et al. 1990 |
| *rbc*L 1F | ATGTCACCACAAACAGAAACTAAAGCA | Olmstead et al. 1992 |
| *rbc*L 1460R | TCCTTTTAGTAAAAGATTGGGCCGAG | Olmstead et al. 1992 |
| *matK* KIM 1R | ACCCAGTCCATCTGGAAATCTTGGTTC | Kim KJ 2009, unpublished |
| *matK* KIM 3F | CGTACAGTACTTTTGTGTTTACGAG | Kim KJ 2009, unpublished |

S3.3 Taxa, herbarium voucher numbers and GenBank accession numbers.

| **Taxa** | | **Voucher** | **GenBank** | | |
| --- | --- | --- | --- | --- | --- |
|  |  |  | **ITS** | *rbc*L | *matK* |
| *Acaena alpina* Poepp. ex Walp. | 184996 | | MH781148 | ON542548 | ON542515 |
| *Acaena pinnatifida* Ruiz & Pav. |  | | MH781149 | MF963399 | MF963761 |
| *Acaena splendens* Hook. & Arn | 184995 | | MH781150 | ON542549 | ON542516 |
| *Adesmia glomerula* Clos |  | | MH781156 | MZ198417 |  |
| *Adesmia montana* Phil. |  | | MH781157 | MZ198416 |  |
| *Adesmia schneideri* Phil | 185059 | | MH781159 | ON542550 | ON542517 |
| *Alstroemeria exserens* Meyen |  | | MH792061 | JQ404670 |  |
| *Anarthrophyllum cumingii* (Hook. & Arn.) F.Phil. | 184986 | | MH781160 | ON542551 | ON542518 |
| *Azorella madreporica* Clos | 185063 | | MH781165 | MZ198403 | ON542519 |
| *Azorella ruizii* G.M.Plunkett & A.N.Nicolas | 191861 | | KM671870 | DQ133815 | ON542520 |
| *Berberis empetrifolia* Lam. |  | |  | MZ198467 |  |
| *Calceolaria arachnoidea* Graham | 190301 | | ON521128 | AY423108 | ON542521 |
| *Cerastium arvense* Cham. & Schltdl. | 184989 | |  | ON542552 | ON542522 |
| *Chaetanthera euphrasioides* Reiche | 191803 | | DQ355866 | ON542553 | ON542523 |
| *Chenopodium philippianum* Aellen |  | | KF709219 |  |  |
| *Chuquiraga oppositifolia* D.Don |  | | EU841151 | EU841109 | EU841332 |
| *Collomia biflora* (Ruiz & Pav.) Brand | 185087 | | MH781180 | ON542554 | HQ116935 |
| *Convolvulus demissus* Choisy |  | | KC528836 | KC529198 | KC529039 |
| *Ephedra chilensis* C.Presl |  | |  | AY492036 | AY492012 |
| *Euphorbia collina* Willd. Ex Ledeb. | 190283 | |  | ON542555 |  |
| *Galium gilliesii* Hook. & Arn. | 190326 | | ON521129 |  |  |
| *Haplopappus anthylloides* Meyen & Walp. | 185055 | | MH781202 | ON542556 | ON542524 |
| *Haplopappus schumannii* (Kuntze) G.K.Br*.* & W.D.Clark | 185092 | | MH781204 | ON542557 | ON542525 |
| *Hordeum comosum* J. Presl |  | | AJ607876 | AY137441 | AB078097 |
| *Hypochaeris clarionoides* (J. Remy) Reiche | 190361 | | ON521130 |  | AF528408 |
| *Jaborosa caulescens* Gillies & Hook. | 185041 | | MH781206 | ON542558 | ON542526 |
| *Latace andina* (Poepp.) Sassone |  | | KF171082 |  |  |
| *Loasa caespitosa* Phil. | 191792/190397 | | ON521131 | ON542559 | ON542527 |
| *Melosperma andicola* Benth. |  | | MH781214 | MZ198432 | AY492153 |
| *Microsteris gracilis* (Hook.) Greene | 184982 | | MH781217 | ON542560 | ON542528 |
| *Montiopsis gilliesii* (Hook. & Arn.) D.I.Ford |  | | DQ090406 |  |  |
| *Mutisia sinuata* Cav. |  | | MH781221 | EU841128 | EU841355 |
| *Nassauvia looseri* Cabrera |  | | MG432167 | EU841132 | EU841358 |
| *Nassauvia pyramidalis* Meyen |  | | MG432171 |  | EU841359 |
| *Nastanthus ventosus* (Meyen) Miers | 185039 | |  |  | ON542529 |
| *Nicotiana corymbosa* J. Remy | 185007 | | MH781227 | ON542561 | ON542530 |
| *Noccaea magellanica* (Pers.) Holub | 185067 | |  |  | ON542531 |
| *Oreopolus glacialis* (Poepp. & Endl.) Ricardi |  | | MH095807 |  |  |
| *Oriastrum chilense* Wedd. | 191823 | | DQ355916 | ON542562 | ON542532 |
| *Oxalis compacta* Gilles | 185002 | | MH781235 | ON542563 | ON542533 |
| *Oxalis squamata* Zucc. | 185011 | | MH781237 | JN587339 | ON542534 |
| *Pappostipa chrysophylla* (E.Desv.) Romasch. |  | | EU489113 |  | EU489194 |
| *Patosia clandestina* Buchenau |  | | AY973514 | U49225 |  |
| *Perezia carthamoides* Hook. & Arn. | 191797 | | FJ979641 | EU841130 | ON542535 |
| *Phacelia secunda* J.F.Gmel. | 191801 | | ON521132 | ON542564 | ON542536 |
| *Poa holciformis* J.Presl |  | | GQ324512 |  |  |
| *Polygonum bowenkampi* Phil. | 185069 | | MH781241 |  | ON542537 |
| *Pozoa coriacea* Lag. | 185016 | | MH781242 | ON542565 | ON542538 |
| *Quinchamalium chilense* Willd. |  | |  | EF464533 | EF464514 |
| *Quinchamalium parviflorum* Phil. |  | | MH781244 |  |  |
| *Rhodolirium montanum* Phil. | 184997 | | MH781245 |  | ON542539 |
| *Rytidosperma violaceum* (E.Desv.) Nicora |  | | EU401404 |  |  |
| *Sanicula graveolens* Poepp. ex DC. | 191804 | | ON521133 | MF963118 | ON542540 |
| *Senecio bustillosianus* J.Remy | 185056 | | MH781248 | ON542566 | ON542541 |
| *Senecio pentaphyllus* Phil. | 185029 | | MH781252 | ON542567 | ON542542 |
| *Sisyrinchium arenarium* Poepp. | 185052 | | MH781255 | ON542568 | ON542543 |
| *Sisyrinchium cuspidatum* Poepp. | 185023 | | MH781256 | ON542569 | ON542544 |
| *Stachys philippiana* Vatke | 185100 | | MH781259 | ON542570 | ON542545 |
| *Taraxacum officinale* F.H.Wigg. |  | | KT249884 | KM361005 | MG947034 |
| *Tetraglochin alata* (Gillies ex Hook. & Arn.) Kuntze | 185051 | | MH781260 | ON542571 | ON542546 |
| *Trisetum preslei* E.Desv. |  | | KU883528 | AY395565 | KU883578 |
| *Tropaeolum polyphyllum* Cav. | 190299 | | AF254041 | AF254043 | ON542547 |
| *Viola atropurpurea* Leyb. | 185060 | | MH781264 | ON542572 |  |
| *Viola philippii* Leybold | 185015 | | MH792062 | ON542573 |  |
| *Ginkgo biloba* L. |  | | EF372233 | AJ235804 | EF468640 |

S3.4. Results of Mantel tests of community composition with environmental variables.

| **Community composition** | **Comparision matrix(ces)** | **Test** | **R** | **p** |
| --- | --- | --- | --- | --- |
| Taxonomic | soil and elevation | Mantel | 0.63 | 0.001 |
| Phylogenetic | soil and elevation | Mantel | 0.3 | 0.019 |
| Functional | soil and elevation | Mantel | 0.51 | 0.001 |
| Spectral | soil and elevation | Mantel | 0.23 | 0.036 |
| Taxonomic | elevation | Mantel | 0.63 | 0.001 |
| Phylogenetic | elevation | Mantel | 0.3 | 0.02 |
| Functional | elevation | Mantel | 0.51 | 0.002 |
| Spectral | elevation | Mantel | 0.23 | 0.021 |
| Taxonomic | soil | Mantel | 0.007 | 0.4 |
| Phylogenetic | soil | Mantel | -0.08 | 0.7 |
| Functional | soil | Mantel | -0.46 | 0.6 |
| Spectral | soil | Mantel | -0.19 | 0.98 |
| Taxonomic | soil and elevation | Partial Mantel | 0.026 | 0.4 |
| Phylogenetic | soil and elevation | Partial Mantel | -0.054 | 0.6 |
| Functional | soil and elevation | Partial Mantel | 0.001 | 0.5 |
| Spectral | soil and elevation | Partial Mantel | -0.18 | 0.9 |
